# Ultrastructural pathology of primary ciliary dyskinesia: report about 125 cases in Germany

**DOI:** 10.1186/1746-1596-6-115

**Published:** 2011-11-24

**Authors:** Dirk Theegarten, Michael Ebsen

**Affiliations:** 1 Institute of Pathology and Neuropathology, University Hospital Essen, University of Duisburg-Essen, Hufelandstrasse 55, 45147 Essen, Germany; 2 Institute of Pathology, MVZ Municipal Hospital, 24105 Kiel, Germany

## Abstract

**Background:**

Primary ciliary dyskinesia (PCD) is a rare genetically induced disorder of cilia inducing mainly respiratory diseases. Transmission electron microscopy (TEM) analysis of ciliary ultrastructure is classically used for diagnosis. We report our experience of TEM investigations in a large series of patients.

**Methods:**

TEM analysis performed of 742 biopsies from patients with suspected PCD was reviewed retrospectively. Ultrastructural defects were analysized further in 125 cases with changes typical for PCD.

**Results:**

In 18.1% of patients diagnosis of PCD was made because of morphological alterations, in 68.2% secondary changes were seen. In 13.7% material was not feasible for analysis. Mostly defects of dynein arms were detected in PCD (96.8%). In particular defects of the inner arms (51.2%) and combined dynein defects (37.6%) were found. Total loss of dynein arms was dominant. Only in 3.2% deficiencies of central structures were found alone. Associated situs inversus or dextracardia was reported clinically in 21.4%.

**Conclusions:**

TEM analysis is possible in most patients and a useful tool for diagnosis of PCD. Functional and genetic analysis should be done additionally. Registers should be installed to collect all available informations and push further research.

## Background

Cilia are ancient evolutionarily conserved organelles that typically project from the apical surface of cells. Their biological roles include whole-cell locomotion, movement of fluid, chemo-, mechano- and photosensation and sexual reproduction. Defects in cilia are associated with a growing number and wide range of human diseases, which in the whole are called ciliopathies [1].

Historically the disease has been described as immotile cilia syndrome by BA Afzelius [2]. Primary ciliary dyskinesia (PCD) is a predominantly autosomal recessive inherited disorder. Estimation of prevalence is difficult and given between 1:10,000 to 40,000 live births [3,4]. Recurrent and chronic upper and lower respiratory tract infections, and in 40-50% of cases, mirror-image organ arrangement and other forms of heterotaxy are main symptoms [4-6]. Symptoms vary according to the age in which diagnosis is made. Diagnosis of PCD requires the presence of the characteristic clinical phenotype and either specific ultrastructural ciliary defects identified by TEM or abnormal ciliary function [3-6]. In a few cases only abnormal function is found aloneside with normal ultrastructure, therefore analysis of ciliary function and TEM are recommended. Guidelines and algorithms have been developed to standardize diagnostic procedures [4,7]. Diagnostic modalities will vary in different countries. A recent questionnaire survey of the European Respiratory Society in Austria reported that TEM analysis was done in 73% of all PCD patients [8].

The axoneme of motile cilia is composed of nine peripheral doublet microtubules with attached outer and inner dynein arms (ODA and IDA, respectively) and radial spokes, surrounding a central complex (CC) with two central microtubules and the central sheath (so called 9 + 2 tubulus structure) [1]. Cilia consist of over 250 various proteins, making a broad spectrum of defects possible. Genes involved in PCD have been characterized, but not all cases can be described genetically [4]. PCD seems to be linked with malfunctioning in adenosine triphosphate metabolism [9,10]. In TEM analysis different defects are seen in PCD, but only some larger series describing these defects exist [11-18]. Sometimes only case reports are given [9,19]. Therefore we reviewed our cases with ultrastructural diagnosis of PCD retrospectively to compare the results with other studies.

## Methods

All specimens sent to the Institutes of Pathology in Bochum (2002-2006), Essen and Kiel (2006-2009) for TEM analysis because of suspected PCD were analysed retrospectively. Specimens were taken by paediatrics or respiratory physicians and obtained from nasal or tracheobronchial mucosa. Brushings or biopsies were immersed in buffered 2.5% glutaraldehyde and processed as usually [20]. Semithin sections were done for prior selection of suitable blocks. Ultrathin sections were examined at at least 60,000 fold magnification. In each case at least 50 transverse ciliary sections of different cells were investigated. Ciliary abnormalities in over 20% of available axonema were required for diagnosis of primary defects. Ultrastructural defects of dynein arms were divided in cases with total loss or rudimental structure of IDA or ODA.

## Results

### TEM feasibility

Totally 742 specimens were analysed. 102 (13.7%) of these were not suitable for further investigations. In 95.1% no cilia were found at all (in 11.8% metaplasia, in 5.9% no material), and in 4.9% only a few cells with cilia were seen, In 506 (68%) cases only secondary changes due to respiratory infections were detected. Secondary changes were seen in a variable extend and consisted of plebs, numerical aberrations of microtubuli in some cilia, and compound cilia. But in 16 (3.2%) of these cases situs inversus, Kartagener Syndrome or dextrocardia was reported clinically. In 11.1% of the specimens a repeated investigation was recommended because only a relatively small number of cilia were seen. In 134 (18%) cases primary changes were found, ultrastructural defects were documented in detail in 125 patients. Mostly nasal brushings were presented (64%).

### Geographics

Brushings or specimens were recruited from patients who were visiting paediatrics or respiratory physicians in different states in Germany. Most cases of PCD (n = 125) were diagnosed in Northrhine-Westphalia (60.8%). Others were found in Schleswig-Holstein (11.2%), Lower Saxony (8.8%), Hesse (8.8%), Berlin (4.8%), Baden-Württemberg (2.4%), Bremen (1.6%), Hamburg (0.8%), and Rhineland-Palatinate (0.8%).

### Clinical features in PCD

Median of age in PCD was 7.7 (range: 0.1 - 50) years. 59.2% of patients were male, 40.8% female. According to clinical informations given together with the specimens in 103 (82.4%) cases chronic infections (upper and lower respiratory tract or otitis) were dominant (85.4%). In 22 (21.4%) patients situs inversus or dextrocardia was reported. Results of ciliary motility analysis were communicated in 34 (27.2%) cases, abnormal findings were dominant (85.3%). Additional surgical interventions were done in three patients (two middle lobe resections because of destroyed lobe, one double lung transplantation because of bronchiectasis).

### Ultrastructural defects in PCD

**Figure 1:**
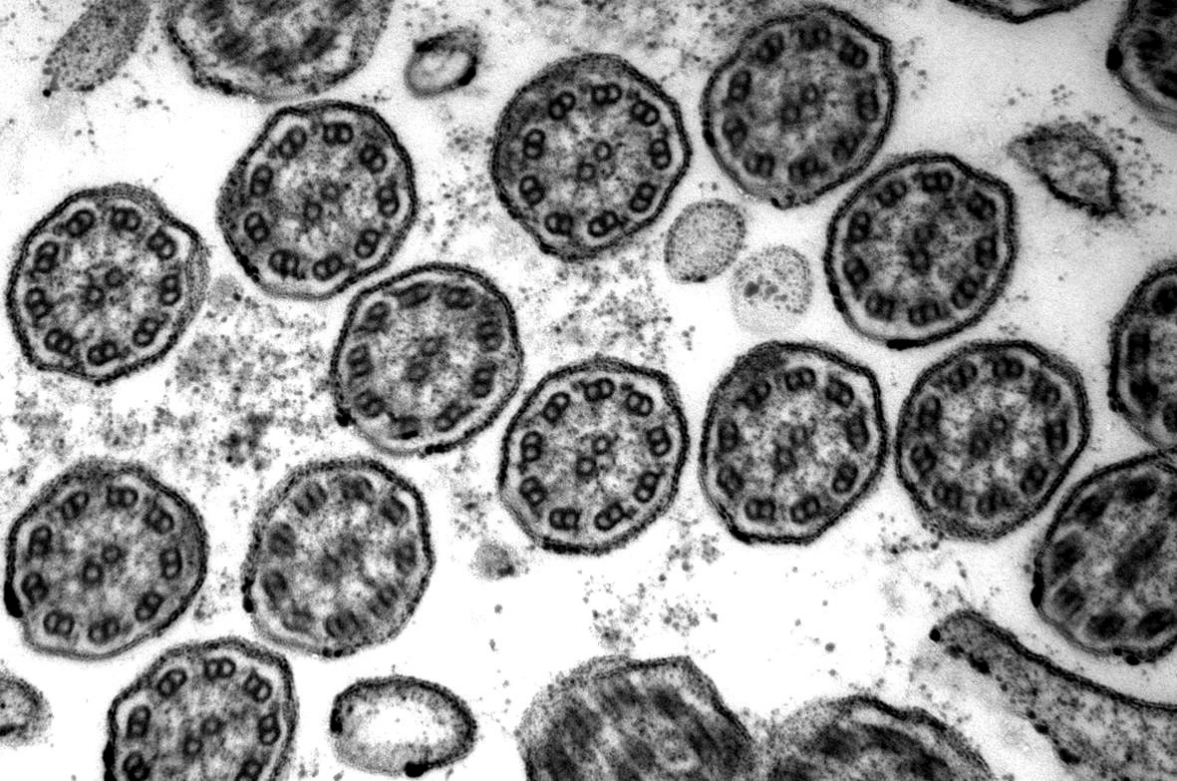
**Defect of inner dynein arms**. Loss of inner dynein arms is the ultrastuctural alteration mostly seen (original magnification × 85,000).

**Figure 2:**
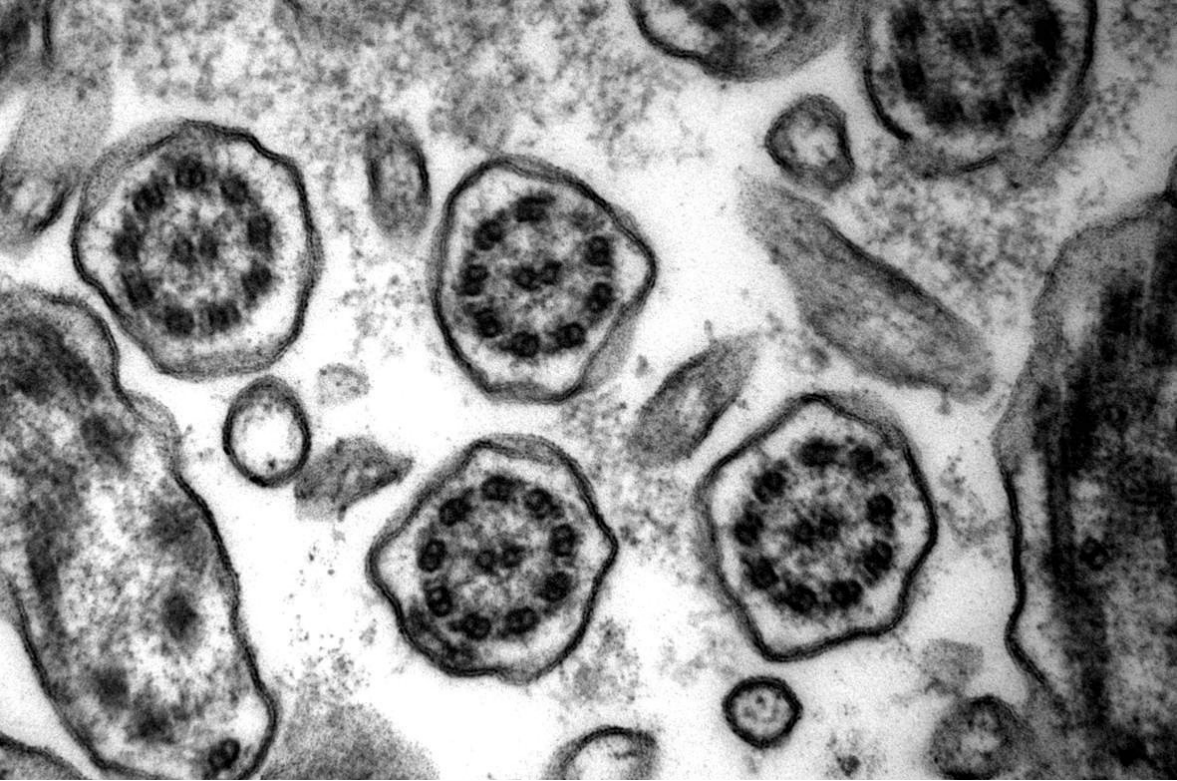
**Defect of inner dynein arms and secondary changes**. Secondary changes with compound cilia (left and right) and plebs are often found in variable extends (original magnification × 85,000).

**Figure 3:**
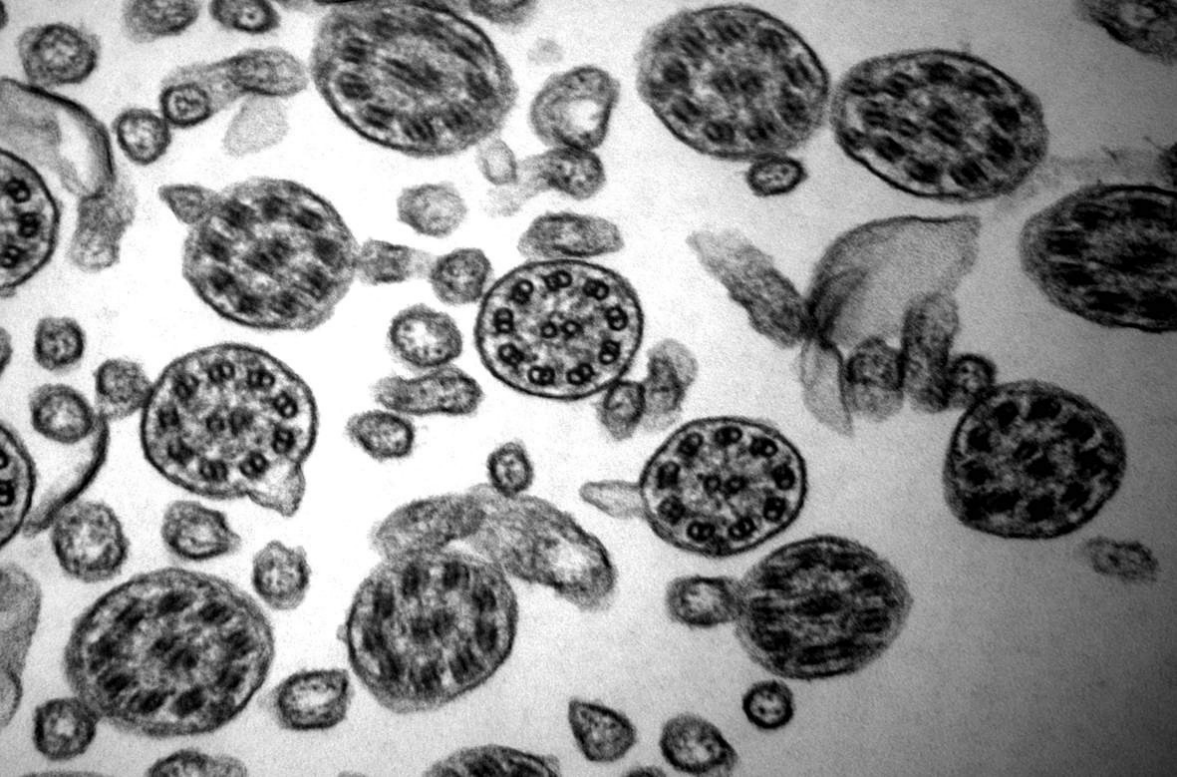
**Defect of outer and inner dynein arms**. Combined defects of outer and inner dynein arms is the lesion on the second place (original magnification × 85,000).

**Figure 4:**
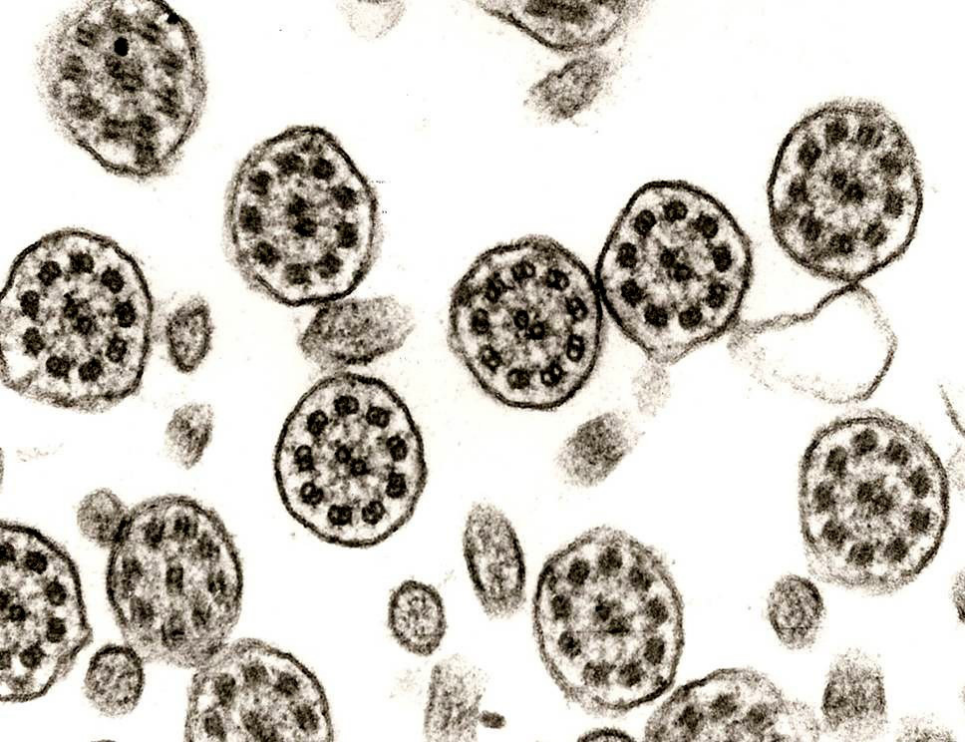
**Defect of outer dynein arms**. Isolated defect of outer dynein arms is only detected in some cases (original magnification × 85,000).

In 96.8% defects of dynein arms (DA) were seen (Table 1). Defects of the inner dynein arms (IDA) were dominant (92%). Isolated defects of the IDA were found in 51.2% [Figure 1, 2]. Combined IDA and outer dynein arm (ODA) defects were detected in 37.8% [Figure 3]. Isolated defects of the ODA were only seen in 6 cases (4.8%) [Figure 4]. Total loss of the DA was dominant, rudimentary structures were detected less frequently (Table 2). Complete missing was found in 75.2% (IDA) vs. 66.6% (ODA).

**Table 1:** Ultrastructural defects in PCD

Ultrastructural defect(s)	numbers (n = 125)	percentage
Inner dynein arms	64	51.2
Outer and inner dynein arms	47	37.8
Outer dynein arms	6	4.8
Central tubuli	2	1.6
Outer and inner dynein arms and radial spokes	2	1.6
Central double tubuli	1	0.8
Outer and inner dynein arms and central tubuli	1	0.8
Central tubuli and radial spokes	1	0.8
Inner dynein arms and tubuli	1	0.8

Mainly defects of inner and outer dynein arms are seen.

**Table 2:** Defects of dynein arms

Defect(s) of dynein arms	totally missing	rudimentary
Inner dynein arms	48 (75.2%)	16 (24.8%)
Outer dynein arms	4 (66.6%)	2 (33.3%)

Defects vary from total loss to rudimentary structure.

**Figure 5:**
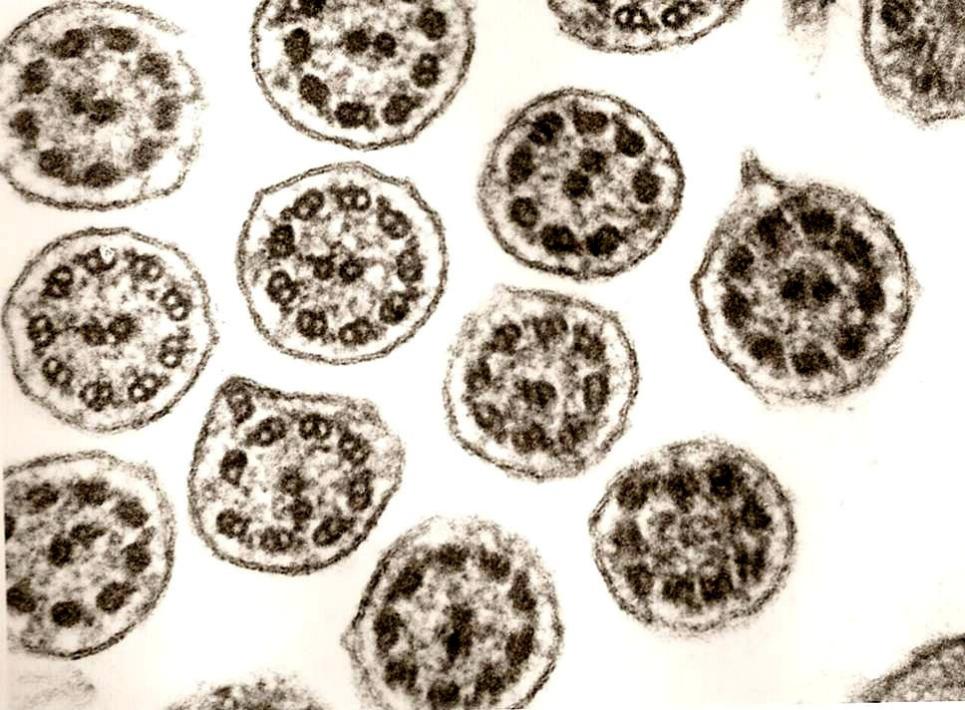
**Defect of radial spokes**. This defect causes irregular orientation of the outer double tubuli because of missing fixation (original magnification × 85,000).

**Figure 6:**
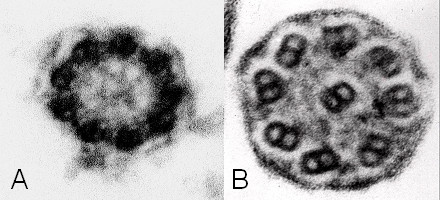
**Single cilia view**. In one case central tubuli were not found, dynein arms can not be evaluated in this picture (A, original magnification × 140,000). Combined defect with missing dynein arms and loss of outer double tubuli is seen (B, original magnification × 140,000).

Only in 4 cases (3.2%) deficiencies of central structures were found alone [Figure 5, 6A]. In all of these the central tubulus pair was missing, in one case additionally radial spokes were not seen. A combination of DA and central defects was seen in 4 cases, in 2 cases three different defects were combined [Figure 6B].

In ten cases (8%) diagnosis of probable PCD was made, because of the limited number of cilia detectable in ultrathin sections. Secondary changes were often seen additionally [Figure 2]. Mostly plebs, loss of one outer membrane, compound cilia and microtubular defects were found.

## Discussion

PCD is a rare genetic disease, therefore only some studies with a larger number of patients exist. In our study material was not feasible for analysis in 13.7%. Papon et al. [18] report a much more higher rate of 28.6%. Feasibility can be improved by using fluorescence bronchoscopy, but in pediatrics mostly unaimed brushings are taken. Some studies with a smaller number of cases (≤ 25) show difficulties to find ultrastructural defects (no dynein arm defects in 42.9%) in PCD [21] or a high percentage (41.2%) of only possible PCD cases [22]. In the largest study (n = 245 PCD cases) the rate of questionable defects was only 3.3% [18]. Ten cases (8%) in our study were classified as probable PCD because of the limited number of detectable cilia. Our results on TEM diagnosis of PCD base on own ultrastructural investigations and clinical informations given with the specimens. Review of clinical records was not possible, this may explain the mismatches with the literature concerning the percentage of cases with situs inversus, which was relatively low [see Additional file 1]. Gender differences do not seem to exist, although a small male predominance was seen in our study. Studies of PCD are all covering a geographical region or country, which may also reflect a special genetic background with different results [see Additional file 1]. In studies with only a smaller number of cases dynein defects were not differentiated [13,16]. Main differences compared with other studies are found in the percentage of cases with defects of ODA and non-DA defects, which were relatively low in our study.

Disorientation of cilia can be found in PCD and was initially supposed to be a form of defect able to induce PCD [23]. Further studies support that these are secondary changes only [24].

Studies on ciliogenesis in cell culture are practised by Jorissen et al. [25], but do not seem to be a widely practised procedure. According to our experience computer-assisted analysis is not necessary [26].

Secondary changes of cilia are known in PCD, as a results of repeated infections and inflammation and sometimes also in healthy subjects [11]. Defects belonging to this category are plebs, loss of one outer membrane, compound cilia and numerical microtubular defects. In cases of severe secondary changes and infections repeated biopsies in an infect-free interval should be requested. In 16 (3.2%) cases with only secondary changes in TEM analysis situs inversus, Kartagener Syndrome or dextrocardia were reported clinically. This is in accordance to other studies. Papon et al. [18] found 28 (5.2%) patients with situs inversus in a group with normal ciliary ultrastrucure (total = 533). In these cases we generally recommended analysis of ciliary beating frequency and pattern as well as repeated TEM investigation. Functional analysis should be done generally to detect cases with normal ultrastructure and to correlate results of both investigations [27]. Recently O'Callaghan et al. have demonstrated, that in cases with IDA defects in TEM analysis spontaneous restitution can occur. Therefore repeated testing is required [28]. Genetic analysis should be done, but does not cover all possible defects today [4]. Therefore PCD registers with all available information should be installed to push further knowledge. Quality control should be practised concerning functional and ultrastructural investigations.

## Conclusions

Complete clinical information concerning history and results of functional analysis should be given to the pathologist. Brushings or biopsies not adequate for diagnostics should be repeated.

Functional and genetic analysis should be done as well to gather all available information.

PCD registers with all clinical, functional and pathological findings and biobanks should be installed to push further research.

## Competing interests

The authors declare that they have no competing interests.

## Authors' contributions

ME did all TEM analysis of the specimens in Bochum and Kiel, collected data from these patients and reviewed fotos from the specimens investigated in Essen. DTH did examinations of specimens in Essen, collected and analysed final data and wrote the manuscript. All authors provided fotos and revised the manuscript. All authors read and approved the final manuscript.

## Supplementary Material

Additional file 1 **Clinical and ultrastructural data of other larger studies**. Explanations: S. inv./dex. = Situs inversus/dextrocardia, *combined defects, **with ultrastructural defects, ***according to clinical information. table. Click here for file

## References

[B1] Badano JL Mitsuma N Beales PL Katsanis N The ciliopathies: an emerging class of human genetic disorders Annu Rev Genomics Hum Genet 2006 7 125 148 10.1146/annurev.genom.7.080505.115610 16722803

[B2] Afzelius BA A human syndrome caused by immotile cilia Science 1976 193 4250 317 319 10.1126/science.1084576 1084576

[B3] Ferkol T Mitchison HM O'Callaghan C Leigh M Carson J Lie H Rosenbluth D Brody SL Current issues in the basis mechanisms, pathophysiology, diagnosis and management of primary ciliary dyskinesia Eur Respir Mon 2006 37 291 313

[B4] Barbato A Frischer T Kuehni CE Snijders D Azevedo I Baktai G Bartoloni L Eber E Escribano A Haarman E Hesselmar B Hogg C Jorissen M Lucas J Nielsen KG O'Callaghan C Omran H Pohunek P Strippoli MP Bush A Primary ciliary dyskinesia: a consensus statement on diagnostic and treatment approaches in children Eur Respir J 2009 34 1264 1276 10.1183/09031936.00176608 19948909

[B5] Bush A Cole P Hariri M Mackay I Phillips G O'Callaghan C Wilson R Warner JO Primary ciliary dyskinesia: diagnosis and standards of care Eur Respir J 1998 12 982 988 10.1183/09031936.98.12040982 9817179

[B6] Coren ME Meeks M Morrison I Buchdahl RM Bush A Primary ciliary dyskinesia: age at diagnosis and symptom history Acta Paediatr 2002 91 667 669 10.1111/j.1651-2227.2002.tb03299.x 12162599

[B7] Bush A Chodhari R Collins N Copeland F Hall P Harcourt J Hariri M Hogg C Lucas J Mitchison HM O'Callaghan C Phillips G Primary ciliary dyskinesia: current state of the art Arch Dis Child 2007 92 1136 1140 10.1136/adc.2006.096958 17634184 PMC2066071

[B8] Lesic I Maurer E Strippoli MP Kuehni CE Barbato A Frischer T Primäre Ziliendyskinesie in Österreich Wien Klin Wochenschr 2009 121 616 622 10.1007/s00508-009-1197-4 19921128

[B9] Popper H Jakse R Diagnose und Aspekte der Pathogenese des Kartagener-Syndromes an Hand von Nasenschleimhautbiopsien Wien Klin Wochenschr 1982 94 370 372 6216669

[B10] Milara J Armengot M Mata M Morcillo EJ Cortijo J Role of adenylate kinase type 7 expression on cilia motility: possible link in primary ciliary dyskinesia Am J Rhinol Allergy 2010 24 181 185 10.2500/ajra.2010.24.3468 20537283

[B11] de Iongh RU Rutland J Ciliary defects in healthy subjects, bronchiectasis, and primary ciliary dyskinesia Am J Respir Crit Care Med 1995 151 1559 1567 7735615 10.1164/ajrccm.151.5.7735615

[B12] Santamaria F de Santi MM Grillo G Sarnelli P Caterino M Greco L Ciliary motility at light microscopy: a screening technique for ciliary defects Acta Paediatr 1999 88 853 857 10.1111/j.1651-2227.1999.tb00061.x 10503685

[B13] Felix H Holzmann D Function and ultrastructure of cilia in primary ciliary dyskinesia Schweiz Med Wochenschr 2000 130 699 704 10846764

[B14] Noone PG Leigh MW Sannuti A Minnix SL Carson JL Hazucha M Zariwala MA Knowles MR Primary ciliary dyskinesia: diagnostic and phenotypic features Am J Respir Crit Care Med 2004 169 459 467 14656747 10.1164/rccm.200303-365OC

[B15] Carlén B Stenram U Primary ciliary dyskinesia: a review Ultrastruct Pathol 2005 29 217 220 10.1080/01913120590951220 16036877

[B16] Plesec TP Ruiz A McMahon JT Prayson RA Ultrastructural abnormalities of respiratory cilia: a 25-year experience Arch Pathol Lab Med 2008 132 1786 1791 18976016 10.5858/132.11.1786

[B17] Stannard WA Chilvers MA Rutman AR Williams CD O'Callaghan C Diagnostic testing of patients suspected of primary ciliary dyskinesia Am J Respir Crit Care Med 2010 181 307 314 10.1164/rccm.200903-0459OC 19910612

[B18] Papon JF Coste A Roudot-Thoraval F Boucherat M Roger G Tamalet A Vojtek AM Amselem S Escudier E A 20-year experience of electron microscopy in the diagnosis of primary ciliary dyskinesia Eur Respir J 2010 35 1057 1063 10.1183/09031936.00046209 19840971

[B19] Markmann HU Matthus J Das primäre ziliare Dyskinesiesyndrom. Ultrastrukturelle Beobachtungen bei einem Zwillingspaar Pathologe 1990 11 80 84 2330351

[B20] Theegarten D Anhenn O Hotzel H Wagner M Marra A Stamatis G Mogilevski G Sachse K A comparative ultrastructural and molecular biological study on Chlamydia psittaci infection in alpha-1 antitrypsin deficiency and non-alpha-1 antitrypsin deficiency emphysema versus lung tissue of patients with hamartochondroma BMC Infect Dis 2004 4 38 10.1186/1471-2334-4-38 15383149 PMC521078

[B21] Armengot M Milara J Mata M Carda C Cortijo J Cilia motility and structure in primary and secondary ciliary dyskinesia Am J Rhinol Allergy 2010 24 175 180 10.2500/ajra.2010.24.3448 20537282

[B22] Sirvanci S Seda Uyan Z Ercan F Karadag B Ersu R Karakoc F Dagli E San T Quantitative analysis of ciliary ultrastructure in patients with primary ciliary dyskinesia Acta Histochem 2008 110 34 41 10.1016/j.acthis.2007.05.006 17698172

[B23] Rayner CF Rutman A Dewar A Greenstone MA Cole PJ Wilson R Ciliary disorientation alone as a cause of primary ciliary dyskinesia syndrome Am J Respir Crit Care Med 1996 153 1123 1129 8630555 10.1164/ajrccm.153.3.8630555

[B24] Jorissen M Willems T The secondary nature of ciliary (dis)orientation in secondary and primary ciliary dyskinesia Acta Otolaryngol 2004 124 527 531 10.1080/00016480410016270 15224888

[B25] Jorissen M Willems T Van der Schueren B Verbeken E De Boeck K Ultrastructural expression of primary ciliary dyskinesia after ciliogenesis in culture Acta Otorhinolaryngol Belg 2000 54 343 356 11082771

[B26] Escudier E Couprie M Duriez B Roudot-Thoraval F Millepied MC Prulière-Escabasse V Labatte L Coste A Computer-assisted analysis helps detect inner dynein arm abnormalities Am J Respir Crit Care Med 2002 166 1257 1262 10.1164/rccm.2111070 12403696

[B27] Chilvers MA Rutman A O'Callaghan C Ciliary beat pattern is associated with specific ultrastructural defects in primary ciliary dyskinesia J Allergy Clin Immunol 2003 112 518 524 10.1016/S0091-6749(03)01799-8 13679810 PMC7126607

[B28] O'Callaghan C Rutman A Williams GM Hirst RA Inner dynein arm defects causing Primary Ciliary Dyskinesia: Repeat testing required Eur Respir J 2011 38 603 607 10.1183/09031936.00108410 21406509

